# Mouse model of anti-RANKL discontinuation reveals reduced bone mass and quality through disruption of bone remodeling

**DOI:** 10.1038/s41413-025-00433-0

**Published:** 2025-05-28

**Authors:** Koji Ishikawa, Soji Tani, Nobuhiro Sakai, Yoshifumi Kudo, Hideyo Horiuchi, Hiromi Kimura-Suda, Masamichi Takami, Mayumi Tsuji, Katsunori Inagaki, Yuji Kiuchi, Takako Negishi-Koga

**Affiliations:** 1 Department of Orthopaedic Surgery, School of Medicine, Showa University, Tokyo, Japan; 2 Department of Pharmacology, School of Medicine, Showa University, Tokyo, Japan; 3 Department of Dental Education, School of Dentistry, Showa University, Tokyo, Japan; 4 Graduate School of Science and Engineering, Chitose Institute of Science and Technology, Hokkaido, Japan; 5 Department of Applied Chemistry and Bioscience, Faculty of Science and Technology, Chitose Institute of Science and Technology, Hokkaido, Japan; 6 Department of Pharmacology, School of Dentistry, Showa University, Tokyo, Japan; 7 Department of Community Medicine and Research for Bone and Joint Diseases, Juntendo University Graduate School of Medicine, Tokyo, Japan; 8 Department of Pathophysiology for Locomotive Diseases, Juntendo University Graduate School of Medicine, Tokyo, Japan; 9 Department of Medicine for Orthopaedics and Motor Organ, Juntendo University Graduate School of Medicine, Tokyo, Japan

**Keywords:** Osteoporosis, Osteoporosis

## Abstract

The discontinuation of denosumab [antibody targeting receptor activator of nuclear factor kappa B ligand (RANKL)] therapy may increase the risk of multiple vertebral fractures; however, the underlying pathophysiology is largely unknown. In patients who underwent discontinuation after multiple injections of denosumab, the levels of tartrate-resistant acid phosphatase 5b increased compared to pretreatment levels, indicating a phenomenon known as “overshoot.” The rate of decrease in bone mineral density during the withdrawal period was higher than the rate of decrease associated with aging, suggesting that the physiological bone metabolism had broken down. Overshoot and significant bone loss were also observed in mice receiving continuous administration of anti-RANKL antibody after treatment was interrupted, resembling the original pathology. In mice long out of overshoot, bone resorption recovered, but osteoblast numbers and bone formation remained markedly reduced. The bone marrow exhibited a significant reduction in stem cell (SC) antigen 1- and platelet-derived growth factor receptor alpha-expressing osteoblast progenitors (PαS cells) and alkaline phosphatase-positive early osteoblasts. Just before the overshoot phase, the osteoclast precursor cell population expands and RANKL-bearing extracellular vesicles (EVs) became abundant in the serum, leading to robust osteoclastogenesis after cessation of anti-RANKL treatment. Thus, accelerated bone resorption due to the accumulation of RANKL-bearing EVs and long-term suppression of bone formation uncoupled from bone resorption leads to the severe bone loss characteristic of denosumab discontinuation.

## Introduction

Osteoporosis, which is characterized by progressive bone loss and deterioration of the bone microarchitecture, is an enormous and growing public health problem. Bone homeostasis is maintained throughout life by continuous remodeling that depends upon a tightly controlled balance between bone resorption by osteoclasts and bone formation by osteoblasts. During healthy bone remodeling, bone formation is controlled to occur following bone resorption. When this control, called coupling mechanism, breaks down and bone resorption becomes excessive compared to bone formation, osteoporosis is caused.^1,2^ Several of the effective therapeutic agents for osteoporosis, such as denosumab and bisphosphonates, have been developed with a focus on their anticatabolic effects, whereas teriparatide and romosozumab were developed with a focus on their pro-anabolic effects.^3^ Yet despite the remarkable achievement of pro-anabolic agents, the therapeutic window for these agents is limited to only a few years.^3^ Thus, the long-term treatment of osteoporosis requires sequential switching between different agents, including antiresorptive drugs, necessitating a detailed understanding of the characteristics and conditions of drug use.

Denosumab is a fully human monoclonal antibody that targets receptor activator of nuclear factor kappa B ligand (RANKL),^4,5^ blocking its interaction with its receptor RANK on osteoclast precursor cells, thus inhibiting osteoclastogenesis.^1^ Denosumab is currently widely used for the treatment of osteoporosis and rheumatoid arthritis as well as the prevention of skeletal-related events in patients with solid tumors. The long-term efficacy and safety of denosumab in the treatment of osteoporosis have been established by demonstrating a progressive increase in bone mineral density (BMD) and a low incidence of fracture for up to 10 years.^6^

However, post hoc analyses and numerous case reports have raised concerns that discontinuation of denosumab may cause an increased risk of fracture.^7–10^ Upon discontinuation of denosumab, the bone resorption marker, serum C-terminal telopeptide of type I collagen, was markedly increased above pretreatment levels.^11,12^ Histological analyses of patients who stopped taking denosumab also have revealed increases in both osteoclast number and eroded surface.^13,14^ Such rapid disruption of bone metabolism can result in life-threatening hypercalcemia by releasing a surge of calcium into the bloodstream.^7^ Studies also showed an increase in the bone formation marker, serum type I collagen N-terminal propeptide, as well as the restoration of bone formation in bone biopsies, following a rebound in bone resorption, indicating an acceleration of overall bone metabolism.^11,12^ When researchers examined biopsies from patients with a high fracture occurrence, they found that denosumab treatment reduced the survival rate of osteocytes, an effect that persisted even after treatment was discontinued.^14^ These findings suggest that the effects of denosumab treatment are only partially reversible, and that bone remodeling after discontinuation does not fully return to its normal state. In the context of declining adherence,^15^ elucidating the bone pathology after denosumab discontinuation is an important issue for managing denosumab treatment and establishing the timing and method of resuming osteoporosis treatment after discontinuation.

The discontinuation of other anti-osteoporotic agents, including bisphosphonates and teriparatide, also reduces bone mass to pretreatment levels, but it has not been shown to increase the risk of bone fracture, unlike denosumab.^15,16^ Furthermore, a surge of bone resorption (referred to as “overshoot”)^12,17^ does not typically occur after anticatabolic agents are stopped and currently appears to only happen after the discontinuation of denosumab. Denosumab-specific overshoot seems to be explained by differences in the distinguishing features of denosumab and bisphosphonates; the former has rapid reversibility of its antiresorptive effects once it is no longer present in the bloodstream, whereas the latter has long-lasting effects that persist for months or even years after stopping treatment since they are incorporated into the bone matrix due to their high binding affinity for hydroxyapatite. However, there has been little investigation into how denosumab discontinuation causes bone loss at the molecular level.

Here, we developed a mouse model exhibiting overshoot of bone resorption, robust bone loss, and bone fragility, which resemble the human phenotypes after denosumab discontinuation. Mice after discontinuation of the anti-RANKL antibody exhibited not only excessive bone resorption but also long-lasting suppression of bone formation due to a marked reduction of osteoblastic lineage cells even after bone resorption recovered, resulting in a significant decrease in bone mass. Osteoclast precursors as well as RANKL-bearing extracellular vesicles (EVs) accumulated during the anti-RANKL antibody treatment period, and caused an osteoclastic burst after discontinuation. The anti-RANKL antibody suppressed phagocytic clearance of EVs, possibly through binding to RANKL on the EV surface. This study provides new mechanistic insights into how discontinuation of the anti-RANKL antibody causes an osteoclastic burst and disrupts normal bone metabolism, which has important implications for the clinical management of patients with osteoporosis in need of long-term treatment and diverse therapeutic options.

## Results

### Bone loss in mice after discontinuation of anti-RANKL antibody treatment

To identify aberrant bone metabolism after denosumab discontinuation, we first analyzed eight patients (79.9 ± 5.4 years old) who met the inclusion criteria for this study, as described in Fig. S1a (e.g., multiple doses of denosumab and an appropriate period after discontinuation). After discontinuation of denosumab treatment (for more than 10 months), the serum tartrate-resistant acid phosphatase 5b (TRAP-5b) levels in patients were markedly higher than pretreatment levels (Fig. S1b, left), indicating an overshoot. BMD in both the lumbar spine and femoral neck was also decreased to pretreatment levels in these patients (Fig. S1b, middle and right). Compared to the rate of decrease in BMD over a similar time period in the age- and sex-matched untreated controls, those who had discontinued treatment showed a significantly higher rate of decrease (Fig. S1c), despite there being no differences in blood parameters indicative of altered parathyroid or kidney function (Table S1). These findings suggest that BMD decreases independently of physiological bone loss due to age-related osteoporosis, consistent with previous reports.^7–14^

**Fig. 1:**
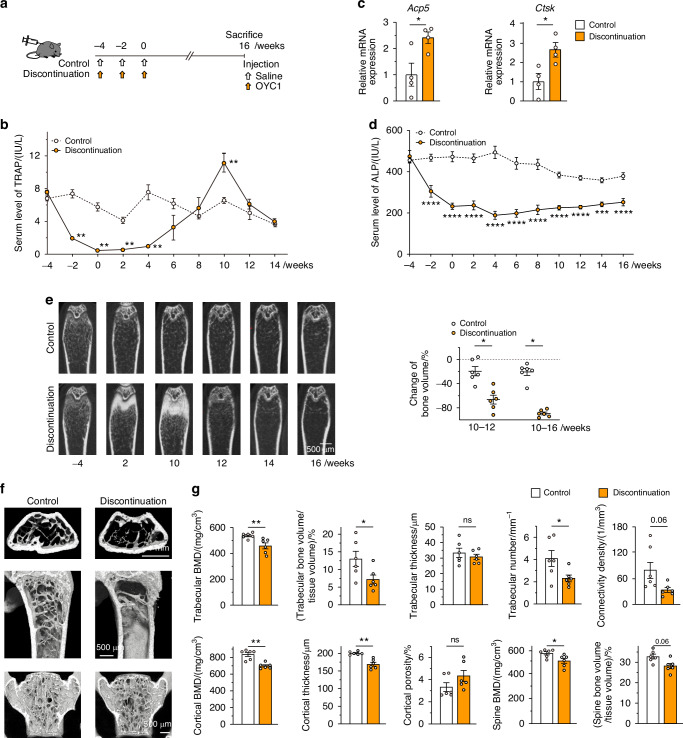
TRAP overshoot and rapid bone loss induced by discontinuation of anti-RANKL treatment in a three-injection mouse model. **a** Schematic of the experimental design for the three-injection mouse model of anti-RANKL discontinuation. **b** Serum TRAP levels over time in anti-RANKL-treated and control mice (*n* = 7). **c** Expression of osteoclastic marker genes (*Acp5, Ctsk*) in the femur during the overshoot period (10 weeks post-treatment, *n* = 4). **d** Serum ALP levels over time (*n* = 8). **e** Bone mass changes over time, with representative in vivo CT images (left) and percent change in bone volume (right, *n* = 6). **f** Representative µCT images of the distal femur and spine at 16 weeks post-treatment (axial, longitudinal, and coronal views). **g** µCT parameters of the femur and spine at 16 weeks post-treatment (*n* = 6). BMD bone mineral density. Data represent at least three independent experiments (mean ± SEM). *P* < 0.05, *P* < 0.01, *P < 0.001, **P < 0.000 1; ns not significant (Student’s *t* test)

Healthy mice were injected with anti-mouse RANKL antibody (OYC1) three times in 2-week intervals (three-injection model; Fig. 1a) to further investigate the effect of discontinuation on bone metabolism, and the bone phenotype was analyzed over time, including before and after the last injection (week 0). Once injected with anti-RANKL antibody, the serum TRAP levels immediately decreased but rapidly increased 6 weeks after the last injection, reaching significantly higher levels than in control mice by 10 weeks (Fig. 1b). The mRNA expression of osteoclastic marker genes [acid phosphatase 5, tartrate resistant (*Acp5*) and cathepsin K (*Ctsk*)] in the tibia and femur was also increased when the serum TRAP levels peaked (at 10 weeks) (Fig. 1c). Serum levels of alkaline phosphatase (ALP) significantly decreased immediately after administration, and continued to remain low for a long time, in contrast to the variability of TRAP levels (Fig. 1d).

In vivo longitudinal computed tomography (CT) analysis revealed rapid bone loss and a significantly higher rate of bone loss at the end of the overshoot period (10–12 weeks; Fig. 1e, left). Notably, the reduction in bone mass was still evident long after discontinuation (10–16 weeks; Fig. 1e, right), indicating that bone loss continues for an unexpectedly long time after TRAP overshoot. These mice exhibited altered bone features after discontinuation as shown by three-dimensional μCT, in which the BMD of the trabecular and cortical bone of the femur as well as the spine was significantly decreased, and trabecular bone volume, trabecular number, and cortical bone thickness were all markedly reduced (Fig. 1f, g). There was no significant difference in the parameter of bone microstructure, connectivity density, but a moderate decrease was observed in mice after discontinuation (Fig. 1g).

When mice received a single injection of anti-mouse RANKL antibody (single-injection model; Fig. S2a), moderate TRAP overshoot was detected, although there was no significant difference at 8 weeks after the injection (Fig. S2b). Bone mass peaked at 4 weeks post-injection and then rapidly declined and continued to decline over a long period (4–12 weeks); although bone mass was not less than that of the control mice (Fig. S2c). The BMD in cortical bone was significantly reduced compared to pretreatment, even in the single-injection mouse model, but no significant difference in trabecular bone was observed (Fig. S2d). Thus, our three-injection mouse model effectively captures the characteristics of patients with denosumab discontinuation.

### Deterioration of bone quality in mice after discontinuation of anti-RANKL antibody treatment

**Fig. 2:**
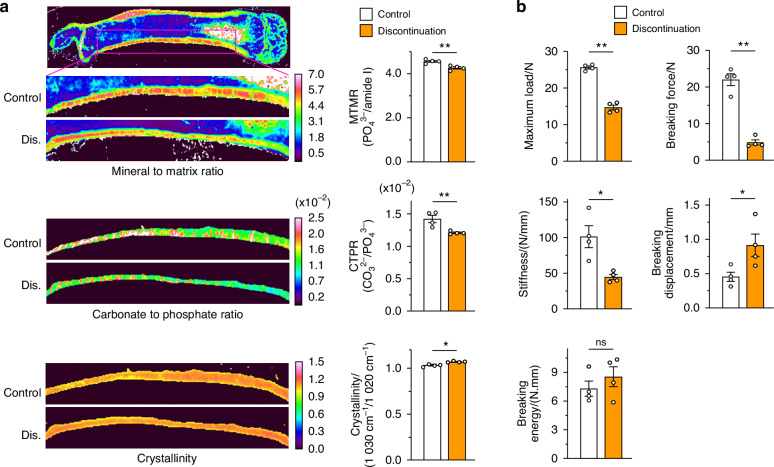
Bone fragility after discontinuation of anti-RANKL treatment in a three-injection mouse model. **a** Representative color mapping images and the parameters obtained from FTIR analysis of the cortical bone of the femur in control and mice with discontinued treatment (Dis) at 16 weeks after the last injection (*n* = 4). **b** Mechanical strength of the femur determined by the three-point bending test at 16 weeks after the last injection (*n* = 4)

Bone fracture is attributed to a combination of multiple factors including the biochemical properties of bone tissue, mechanical loading, and structural characteristics of the bone itself. Chemical mapping by Fourier transform infrared spectroscopy (FTIR) revealed a reduced ratio of mineral to organic matrix and carbonate to phosphate in the cortical area in the three-injection mouse model, indicating an alteration of tissue composition in the bone (Fig. 2a). As the bone’s biomechanical function is reflected in its microstructure and quality, a three-point bending test was performed. After discontinuation of anti-RANKL treatment, the mice exhibited a reduced maximum load, decreased breaking force and stiffness, and increased displacement, suggesting severe bone fragility (Fig. 2b). Taken together, these results showed that discontinuation of anti-RANKL treatment resulted in significant bone fragility caused by bone loss and a deterioration of bone quality.

### Sustained inhibition of osteoblastic bone formation after discontinuation

**Fig. 3:**
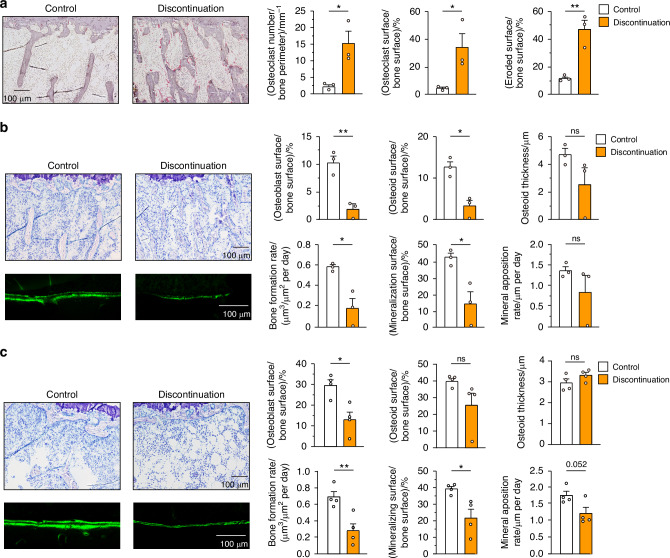
Disruption of bone remodeling after discontinuation of anti-RANKL treatment in a three-injection mouse model. **a** Representative image of TRAP staining (left) and parameters of osteoclastic bone resorption (right) determined by bone morphometric analysis of the proximal tibia of control and Dis mice at 8 weeks after the last injection (*n* = 3). **b** Bone morphometric analysis in the proximal tibia at 8 weeks after the last injection (*n* = 3). Representative image of toluidine blue staining (left upper) and bone formation, as observed by calcein double labeling at an interval of 4 days (left lower, cortical bone). Parameters of osteoblastic bone formation (right). **c** Bone histomorphometric analysis of the proximal tibia at 16 weeks after the last injection (*n* = 4). Representative image of toluidine blue staining (left upper) and bone formation, as observed by calcein double labeling at an interval of 4 days (left lower, cortical bone). Parameters of osteoblastic bone formation (right). All values are representative of at least three independent experiments and are shown as the mean ± SEM. *P < 0.05, **P < 0.01, ns not significant. Student’s *t* test was performed

During TRAP overshoot, the mice that underwent discontinuation of anti-RANKL treatment (8 weeks after the last injection, three-injection model) exhibited a significant increase in osteoclast number, osteoclast surface, and eroded surface (Fig. 3a), consistent with the increase in serum TRAP levels (Fig. 1b). Since bone formation is generally thought to be coupled with bone resorption during the bone remodeling process, this explains reports that anti-RANKL treatment simultaneously reduced levels of bone formation markers as well as resorption.^6,18^ Our bone histomorphometric analysis also revealed that the three-injection mouse model showed a significant decrease in osteoblast surface, osteoid surface and thickness, mineralizing surface, and bone formation rate during the TRAP overshoot period (Fig. 3b). However, interestingly, even after a long period without any exposure to the anti-RANKL antibody (16 weeks after the last injection), these mice exhibited osteoblastic defects such as a decreased osteoblast number and bone formation rate, despite no statistically significant differences in osteoclastic parameters (Fig. 3c and Fig. S3). These results were consistent with the observation that serum ALP levels remained suppressed for a long time after discontinuation (Fig. 1d). Considering our data showing transient TRAP overshoot and long-lasting bone mass reduction, the bone formation appeared to be impaired independently of coupling with bone resorption during this period.

### Specificity of anti-RANKL antibody for causing a decrease in bone mass after discontinuation of treatment

**Fig. 4:**
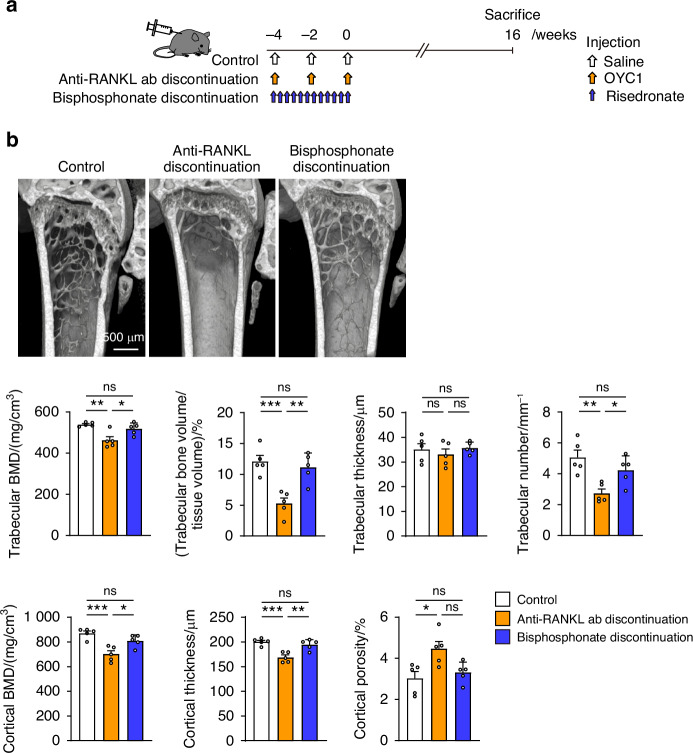
Comparison of the effects of discontinuing anti-RANKL treatment and discontinuing bisphosphonate treatment on bone mass. **a** Schematic of the experimental setting for the mouse model of risedronate discontinuation in mice. **b** Representative images and parameters determined by μCT analysis of the distal femur of mice with anti-RANKL discontinuation (three-injection mouse model), mice with risedronate discontinuation, and control mice at 16 weeks after the last injection (*n* = 5). BMD, bone mineral density. All values are shown as the mean ± SEM. *P < 0.05, **P < 0.01, ***P < 0.001, ns not significant. One-way ANOVA was performed

When patients with osteoporosis discontinue medication, their bone mass often decreases to pretreatment level;^15,16^ however, the overshoot of bone resorption after discontinuation appears to be specific to denosumab. To investigate whether the decrease in bone mass caused by the cessation of bone resorption inhibition is dependent on the properties of the drug used to suppress bone resorption, the effects of anti-RANKL antibody and bisphosphonate were compared. The results showed that when mice were administered the bisphosphonate drug risedronate every other day for 4 weeks and then the treatment was stopped, their bone mass did not decrease to the extent as that in mice that received anti-RANKL antibody and then had their treatment stopped (three-injection model; Fig. 4). The severe bone loss that occurred in the anti-RANKL antibody discontinuation mouse model reproduced the specificity of discontinuing RANKL-targeting treatment, and was similar to the phenomenon observed in patients with osteoporosis who discontinue treatment.

### Effects of anti-RANKL treatment discontinuation on a postmenopausal osteoporosis model

**Fig. 5:**
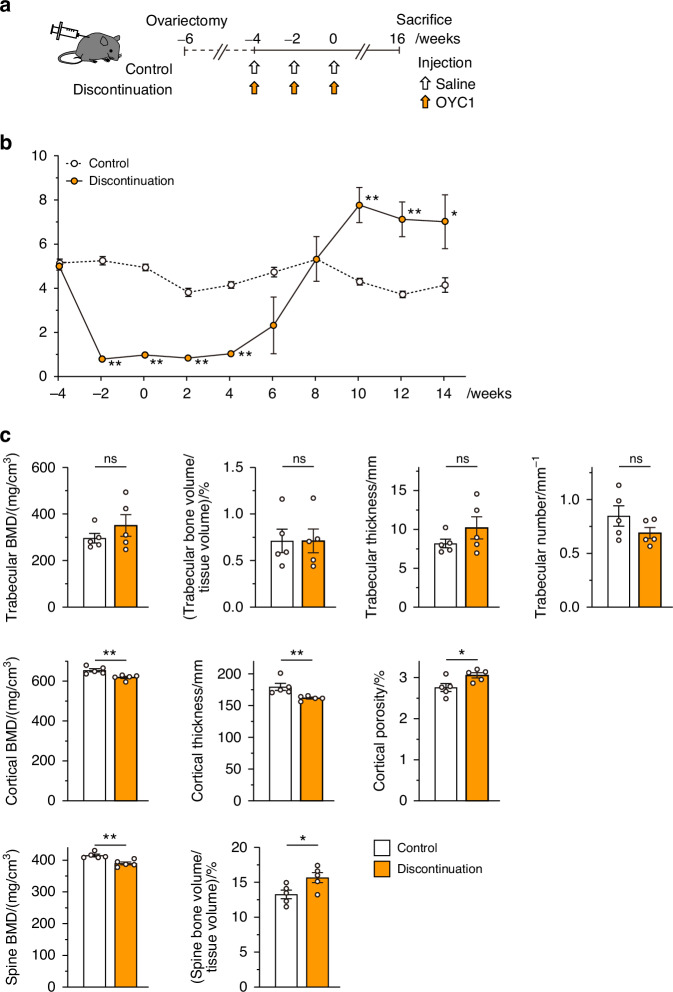
TRAP overshoot and bone mass in ovariectomized (OVX) mice after discontinuation of anti-RANKL treatment in an OVX plus three-injection mouse model. **a** Schematic of the experimental setting for the three-injection OVX mouse model of anti-RANKL discontinuation. **b** Serum TRAP levels over time in anti-RANKL-treated OVX mice (*n* = 6). **c** Parameters determined by μCT analysis of the femur and spine of control and mice with treatment discontinuation at 16 weeks after the last injection. BMD, bone mineral density. All values are representative of at least three independent experiments and are displayed as the mean ± SEM. *P < 0.05, **P < 0.01, ns not significant. Student’s *t* test was performed

The reliability of long-term treatment for osteoporosis is a crucial issue, especially for elderly patients after menopause. In ovariectomized (OVX) mice, a model of postmenopausal osteoporosis (OVX plus three-injection model), it was observed that the serum TRAP overshoot persisted for a longer period after discontinuation of anti-RANKL antibody than in non-OVX mice (three-injection model; Fig. 5a, b). Notably, even a single administration in OVX mice (OVX plus single-injection model) exhibited significantly higher TRAP levels compared to untreated mice (Fig. S2e, f). These results suggested that postmenopausal conditions accelerated the responsiveness to discontinuation. Discontinuation after multiple injections of anti-RANKL antibody (OVX plus three-injection model) led to a reduction of BMD in both the cortical bone and spine, but this decrease was not observed in trabecular BMD (Fig. 5c).

### Accumulation of osteoclast precursor cells in an expanded cell population

**Fig. 6:**
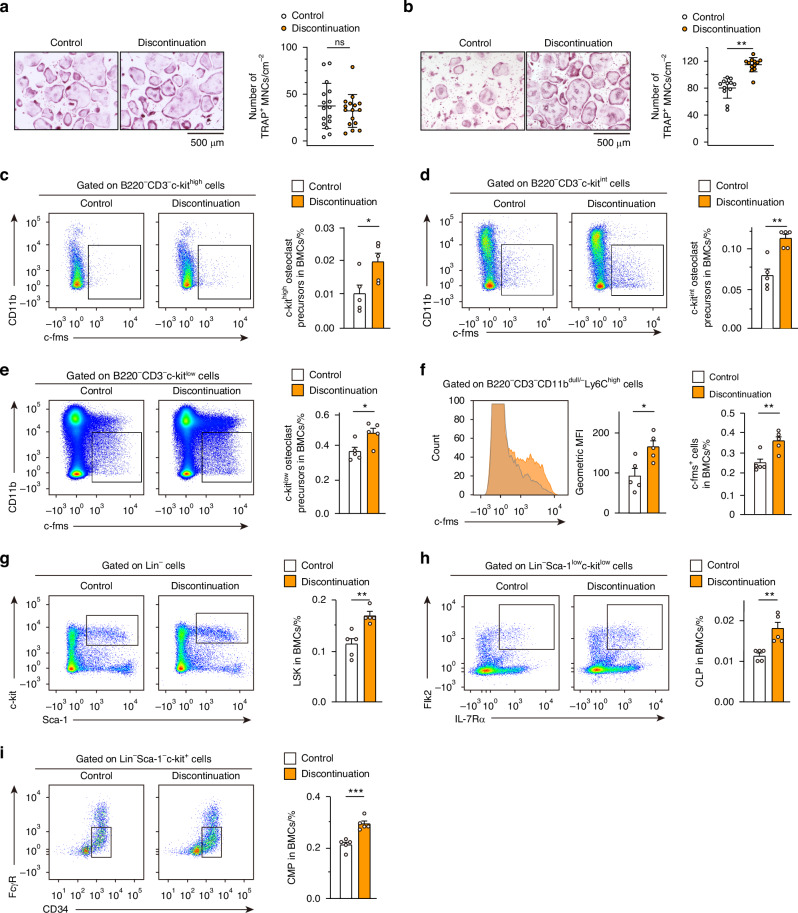
Accumulation of osteoclast precursors and hematopoietic stem cells during anti-RANKL treatment in a three-injection mouse model. **a**, **b** Osteoclastogenesis of bone marrow-derived monocyte/macrophage lineage cells (BMMs) derived from control and anti-RANKL-treated mice at 7 weeks (**a**, *n* = 16) and 2 weeks (**b**, *n* = 12) after the last injection (three-injection model). TRAP-positive multinucleated cells (TRAP^+^ MNCs) were counted. **c**–**e** Percentage of osteoclast precursor cells in control and anti-RANKL-treated mice at 2 weeks after the last injection (*n* = 5). Osteoclast precursors are characterized by the cell surface markers as follows: B220^−^CD3^−^c-kit^high^CD11b^dull^c-fms^+^ cells (**c**), B220^−^CD3^−^c-kit^int^CD11b^dull^c-fms^+^ cells (**d**), and B220^−^CD3^−^c-kit^low^CD11b^dull^c-fms^+^ cells (**e**). **f** Expression of c-fms in the B220^−^CD3^−^CD11b^dull/−^Ly6C^high^ cells in control and anti-RANKL-treated mice at 2 weeks after the last injection (*n* = 5). Representative histogram (left), mean fluorescence intensity (MFI) (middle), and percentage of c-fms^+^ cells in the B220^−^CD3^−^CD11b^dull/−^Ly6C^high^ cells (right) are shown. Percentage of the lineage-negative hematopoietic stem cell population (LSK: Lin^−^Sca-1^+^c-kit^+^) (**g**), as well as common lymphoid progenitors (CLPs) (**h**) and common myeloid progenitors (CMPs) (**i**) in control and anti-RANKL-treated mice at 2 weeks after the last injection (*n* = 5). All data are representative of at least three independent experiments and are shown as the mean ± SEM. *P < 0.05, **P < 0.01, ***P < 0.001. Student’s *t* test was performed

To gain insights into the mechanism underlying bone resorption, the osteoclastogenesis that occurred after discontinuation was investigated using bone marrow monocyte/macrophage lineage cells (BMMs). After an overshoot, there was no difference between the control and anti-RANKL injected mice (three-injection model) in terms of the number of TRAP-positive multinucleated osteoclasts differentiated from the same number of BMMs, suggesting there was no cell-intrinsic alteration related to the osteoclastogenic activity (Fig. 6a). Interestingly, the BMMs derived from anti-RANKL antibody-treated mice before the overshoot phase produced significantly more osteoclasts than BMMs from control mice (Fig. 6b), suggesting that treatment caused an accumulation of osteoclast precursors in vivo.

The cell population characterized as c-kit^+^CD11b^dull/−^c-fms^+^ includes classical precursor cells with high osteoclastogenic potential.^19^ In addition, c-kit^int/low^CD11b^dull/−^c-fms^+^ cells are also osteoclast progenitors that are mainly detected under pathological conditions.^20^ In fact, before the overshoot period (2 weeks after the last injection, three-injection model), mice treated with anti-RANKL antibody had more CD11b^dull/−^c-fms^+^ cells in any subpopulation distinguished by c-kit expression (c-kit^high^, c-kit^int^, and c-kit^low^) (Fig. 6c–e and Fig. S4). On the other hand, high expression of lymphocyte antigen 6 family member C (Ly6C) in myeloid cells is also considered a cell surface marker of osteoclast precursors.^21^ Anti-RANKL antibody-treated mice had higher expression of c-fms in the CD11b^dull/−^Ly6C^high^ population than control mice, suggesting an expansion of osteoclast precursor cells (Fig. 6f and Fig. S4). Although CD11b^dull/−^Ly6C^high^ monocytes increase in response to pathological conditions, including rheumatoid arthritis,^21^ mice treated with anti-RANKL antibody had a comparable number of these cells as the control mice (Fig. S4). These results suggested that, before the overshoot period, the population of cells with osteoclast differentiation potential, characterized by CD11b^dull/−^c-fms^+^, expanded across subpopulations distinguished by c-kit or Ly6C.

Interestingly, we found that cell populations were altered not only in the osteoclast lineage but also in the broader lineage of bone marrow cells. The frequency of a lineage-negative hematopoietic stem cells (HSC) population (LSK: Lin^−^Sca-1^+^c-kit^+^), as well as Lin^−^Sca-1^lo^c-kit^lo^IL-7Rα^+^Flk2^high^ common lymphoid progenitors (CLPs) and Lin^−^Sca-1^−^c-kit^+^IL-7R^−^αCD34^+^FcγR^−^ common myeloid progenitors (CMPs), was increased in mice treated with anti-RANKL antibody (Fig. 6g–i). No significant differences were observed for the inflammatory cytokines tested in this study, with the exception of slight increases in interleukin 6 (IL-6) and granulocyte colony-stimulating factor (G-CSF) (Table S2). It is unlikely that the accumulation of osteoclast precursor cells and alteration of hematopoietic cell populations were caused by inflammatory conditions due to anti-RANKL antibody injection.

### Accumulation of RANKL-bearing EVs during anti-RANKL treatment

The question of what triggers the osteoclast differentiation of accumulated precursors remains unknown. A recent clinical study showed the increased production of serum RANKL in patients who had undergone denosumab discontinuation.^12^ Consistent with this finding, the three-injection mouse model showed an increased ratio of RANKL to serum levels of osteoprotegerin (OPG) (Fig. S5a). Interestingly, however, there was a declining trend in the mRNA expression of *Rankl* [encoded by tumor necrosis factor superfamily member 11 (*Tnfsf11*)] in the bone (Fig. S5b). Furthermore, serum derived from both normal mice and healthy humans had a stimulatory effect on the osteoclastogenesis of normal mouse BMMs and human peripheral blood mononuclear cells (PBMCs), respectively (Fig. S5c, d), suggesting the presence of osteoclastogenic factors in serum.

**Fig. 7:**
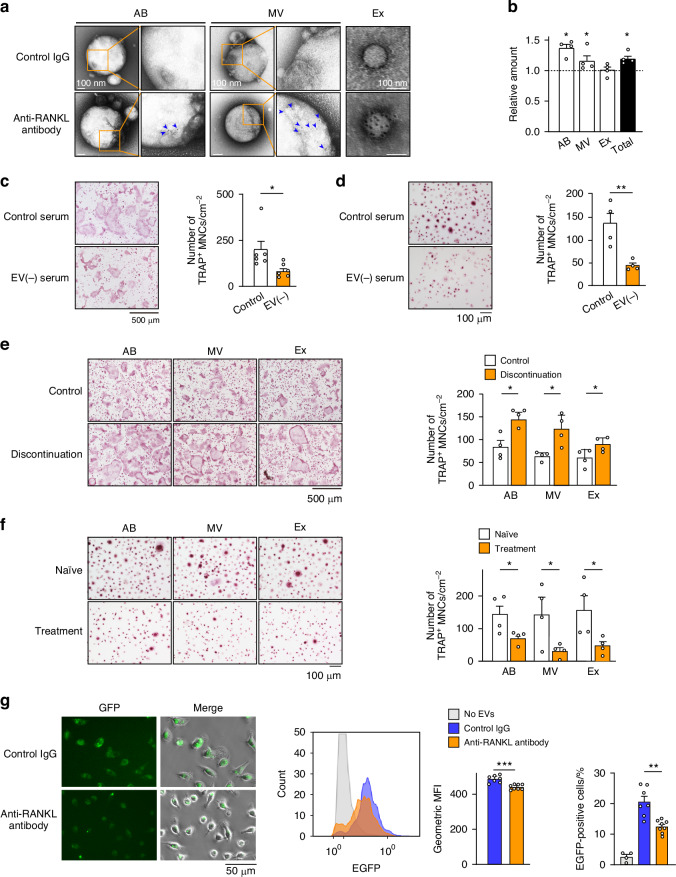
Osteoclastogenesis induced by RANKL-bearing EVs after discontinuation of anti-RANKL treatment in a three-injection mouse model. **a** Representative electron microscopy images of EVs labeled with immunogold for RANKL. EVs were isolated from normal mouse serum (*n* = 6) and labeled with anti-RANKL antibody or control IgG. The orange boxed regions in the left panels of apoptotic bodies (AB) and microvesicles (MV) are shown at higher magnification in the right panels. Arrowheads, immunogold labeling. Ex exosome. **b** Relative protein amount of EVs in anti-RANKL-treated mice compared with untreated mice during the overshoot period (*n* = 4). Effect of EV-depleted normal mouse serum (**c**) and human serum (**d**) on the osteoclastogenesis of mouse BMMs (**c**, *n* = 6) and human PBMCs (**d**, *n* = 4). Representative images and quantification of TRAP-positive multinucleated cells (TRAP^+^ MNCs) are shown. **e** Effect of EVs isolated from the serum of control mice and mice with treatment discontinuation during the overshoot period on the osteoclastogenesis of normal BMMs. Representative images and the number of TRAP^+^ MNCs are shown (*n* = 4). **f** Effect of EVs isolated from the serum of treatment-naïve and patients treated with denosumab on the osteoclastogenesis of healthy human PBMCs. Representative images and the number of TRAP^+^ MNCs are shown (*n* = 4). **g** Effect of anti-RANKL antibody on the uptake of EVs by bone marrow macrophages. Representative images of macrophages phagocytosing EGFP-labeled EVs in the presence of anti-RANKL antibody (*n* = 7) or control IgG (*n* = 8) (left). Representative image and quantification of mean fluorescence intensity (MFI) (middle) and percentage of EGFP-positive cells (right) analyzed by flowcytometry. All data are representative of at least three independent experiments and are shown as the mean ± SEM. *P < 0.05, **P < 0.01, ***P < 0.001, ns not significant. Student’s *t* test was performed

EVs are emerging as important mediators of a range of pathological as well as physiological processes, including the communication of bone cells;^22^ thus, we analyzed them as possible factors that cause TRAP overshoot after anti-RANKL discontinuation. Immunoblotting and immunoelectron microscopy revealed that RANKL was present in all three types of EVs isolated from human and mouse serum, including apoptotic bodies (ABs), microvesicles (MVs), and exosomes (Fig. 7a and Fig. S5e, f). In the same amount of serum, the relative protein levels of ABs and MVs were higher in anti-RANKL-treated mice than untreated mice during the overshoot period (Fig. 7b). The isolated EVs were examined for their contribution to osteoclastogenesis. The ABs and MVs from untreated mice, and all types of EVs from healthy humans effectively stimulated the osteoclastogenesis of BMMs and PBMCs, respectively (Fig. S5g, h). Conversely, in the presence of EV-depleted serum, the osteoclastogenesis of both mouse BMMs and human PBMCs was reduced (Fig. 7c, d). All EVs, even exosomes derived from anti-RANKL antibody-treated mice, exhibited greater induction of osteoclastogenesis than that observed in untreated mice (Fig. 7e). Collectively, the buildup of RANKL in the serum, a portion of which is present on EVs, may have triggered the burst of osteoclastogenesis from the accumulated osteoclast precursors. However, after the discontinuation of bisphosphonate treatment, neither the accumulation of osteoclast precursors nor an increase in the level of serum EVs was observed (Fig. S6). These findings indicated that the overshoot of bone resorption after discontinuation was due to the specific role of the anti-RANKL antibody. Notably, the EVs collected from the serum of patients during denosumab treatment had reduced ability to stimulate osteoclastogenesis compared to EVs collected from treatment-naïve patients with osteoporosis (Fig. 7f), suggesting that the RANKL expressed on EVs did not have a stimulatory role due to a masking effect of denosumab.

EVs are released in various tissues and organs, and are cleared through the phagocytic system in response to diverse circumstances;^23^ however, the details of their release and clearance depending on cell type have not been fully elucidated. We investigated whether anti-RANKL antibody affects the clearance of EVs containing RANKL. When circulating EVs collected from transgenic mice expressing enhanced green fluorescent protein (EGFP-tg mice) were incubated with BMMs from wild-type mice, the EGFP signals were found to be incorporated into the BMMs, and the EGFP intensity was reduced in the presence of anti-RANKL antibody (Fig. 7g). Masking of RANKL on EVs by anti-RANKL antibody might induce resistance to phagocytosis by macrophages, leading to the accumulation of RANKL-carrying EVs. Subsequent disappearance of the anti-RANKL antibody might result in an osteoclastic burst mediated by RANKL-bearing EVs.

### Suppression of osteoblast progenitor cells by anti-RANKL antibody

**Fig. 8:**
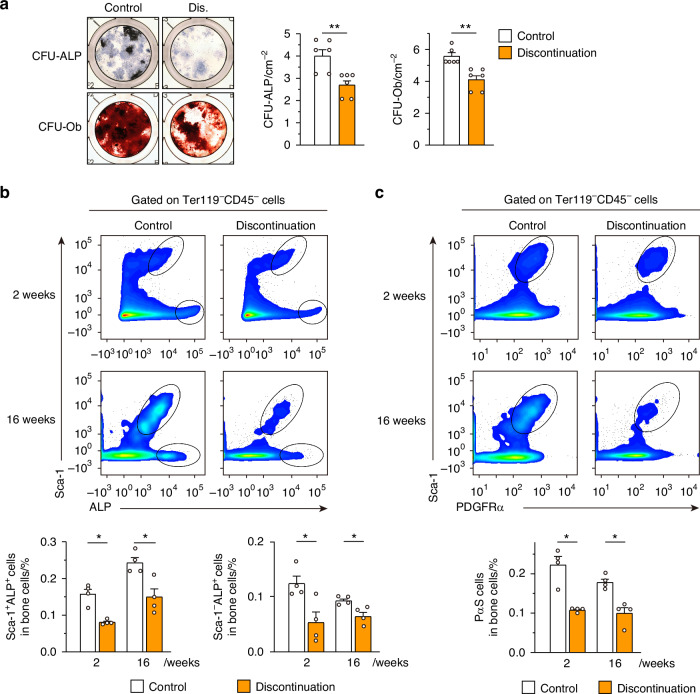
Long-lasting suppression of osteoblastic bone formation after discontinuation of anti-RANKL treatment in a three-injection mouse model. **a** Representative images (left) and quantification (right) of the generation of alkaline phosphate (ALP) positive colony forming units (CFU-ALP) and alizarin-red positive CFUs (CFU-Ob) in bone marrow cells derived from control and mice with treatment discontinuation at 16 weeks after the last injection (*n* = 6). **b** Percentage of osteoblastic progenitor cells characterized by ALP expression in the femur of control mice and mice with treatment discontinuation mice at 2 weeks and 16 weeks after the last injection (*n* = 4). **c** Percentage of osteoblastic progenitors characterized by surface expression of PDGFRα and Sca-1-positive cells (PαS cells) in the femur of control mice and mice with treatment discontinuation (*n* = 4). All data are representative of at least three independent experiments and are shown as the mean ± SEM. *P < 0.05, **P < 0.01. Student’s *t* test was performed

As shown in Fig. 3, the coupling between bone resorption and bone formation was disrupted after discontinuation of anti-RANKL treatment; therefore, the decrease in bone formation appeared to be due to a direct inhibitory mechanism rather than a response to the decrease in bone resorption. To further investigate this point, the osteogenic potential of bone marrow-derived mesenchymal SCs/stromal cells (BMSCs) was examined using the colony formation unit (CFU) assay. In BMSCs derived from mice in the three-injection model at 16 weeks after the last injection, there was a marked decrease in the area of ALP-positive CFUs (CFU-ALP) and Alizarin Red S-positive CFUs (CFU-Ob) (Fig. 8a). In addition, even in the single-injection mouse model and OVX single-injection mouse model, a decrease in CFU-ALP and CFU-Ob in the BMSCs was also observed after discontinuation (Fig. S7a, b). These results suggested that the osteoblast precursor cells in the BMSCs did not lose their ability to differentiate into osteoblasts due to discontinuation, but rather that the number of progenitor cells that could become osteoblasts decreased. Flow cytometry analysis strengthened these results by showing a significant reduction in ALP-positive immature osteoblasts^24^ in the bone marrow of mice after discontinuation (16 weeks after the last injection) (Fig. 8b). Furthermore, BMSCs expressing stem cell antigen-1 (Sca-1) and platelet-derived growth factor receptor alpha (PDGFRα) (PαS cells: Ter119^−^CD45^−^Sca-1^+^PDGFRα^+^),^25^ which give rise to osteoblastic lineage cells, were significantly decreased after discontinuation (Fig. 8c). A three-injection OVX mouse model also showed a significant decrease in ALP-positive immature osteoblasts, but no significant difference was observed in the PαS cell population (Fig. S7c, d). On the other hand, anti-RANKL treatment had no direct effect on either osteoblast differentiation of calvarial cell-derived osteoblasts (Fig. S7e). Together, the results showed that anti-RANKL treatment led to the suppression of bone formation not only through a coupling effect associated with decreased bone resorption but also through decreasing the number of osteoblastic lineage cells; the latter effect in particular may be involved in long-lasting bone mass reduction.

## Discussion

This study established a mouse model of anti-RANKL antibody discontinuation that mimics the severe bone loss observed in patients with osteoporosis after discontinuation of denosumab treatment. In this model, discontinuation of multiple injections of anti-RANKL antibody caused uncoupled bone remodeling, in which bone formation remained suppressed despite the rapid acceleration of bone resorption, resulting in prolonged bone loss and bone fragility. FTIR analysis revealed a reduction in mineral content across the entire cortical bone, including pre-existing bone. These results suggest, for the first time, that discontinuation not only inhibits the formation of new bone but also likely reduces the quality of pre-existing bone due to the long-term disruption of bone metabolism. During the treatment period, expansion of the conventional c-kit^+^CD11b^dull/-^c-fms^+^ osteoclast precursor cell population as well as the kit^-^CD11b^dull/-^c-fms^+^ and CD11b^dull/-^Ly6C^high^ osteoclast precursor cell populations, which are detected in pathological conditions, was observed. The accumulation of precursor cells, along with an increased number of RANKL-bearing EVs, contributed to the osteoclastic burst observed after discontinuation of anti-RANKL treatment. Furthermore, osteoblast progenitors were reduced and did not recover long after the discontinuation of treatment. Thus, even after the transient increase in bone resorption returned to normal levels, the coupling of bone resorption and formation during bone remodeling remained disrupted, leading to long-term bone loss.

Several population-based cohort studies have reported that a longer duration of treatment with denosumab and an increased time interval since the last denosumab injection are associated with greater bone loss and fracture risk. Consistent with these findings, the most severe bone loss was observed in mice that underwent discontinuation after multiple doses in the present study. In addition, consistent with a previous report,^26^ OVX mice showed a significant reduction in cortical and vertebral BMD after discontinuation compared to pretreatment levels. However, this decrease was not observed in trabecular BMD, likely because the volume and BMD of the trabecular bone had already significantly decreased after ovariectomy, so no further decrease in bone mass was observed. Notably, however, OVX mice exhibited TRAP overshoot even after a single dose of anti-RANKL antibody, suggesting that increased turnover in the postmenopausal background was associated with acceleration of bone loss after discontinuation.

Denosumab, similar to the anabolic agent teriparatide, has a reversible effect on BMD after treatment discontinuation, and is markedly different in nature from bisphosphonates, which lose their anti-resorptive effect very slowly due to their affinity for hydroxyapatite. Consistent with a previous report,^27^ the present study also showed that bisphosphonate did not cause bone loss below pretreatment levels, although it gradually decreased after treatment discontinuation. In addition, there was no accumulation of osteoclast precursor cells and EVs in bisphosphonate-treated mice, suggesting that the bone loss following discontinuation of anti-RANKL antibody therapy might have been due to a breakdown of the RANKL-specific biological system. However, the mechanism responsible for RANKL-specific rebound has been unknown until now.

The present study demonstrated that the anti-RANKL antibody suppressed the phagocytosis of EVs by macrophages, possibly resulting in the delayed clearance of RANKL-bearing EVs from the bloodstream and acceleration of osteoclastogenesis through the accumulated EVs. In recent years, extensive studies have revealed that EVs play a role in the physiological and pathological bone microenvironment by carrying a variety of bioactive substances that can influence the behavior of bone cells such as osteoblasts and osteoclasts.^22,28^ RANKL-bearing EVs derived from osteoblast lineages are involved in the communication between osteoclasts and osteoblasts in bone metabolism.^29^ Further clarification is needed regarding the kinetics of RANKL-bearing EVs during anti-RANKL antibody therapy and their contribution to TRAP overshoot. However, they may represent a potential target for artificially controlling TRAP overshoot after treatment discontinuation. In addition, RANKL–RANK signaling also regulates biological systems in various tissues, including not only bone but also lymphatic tissue, mammary glands, blood vessels, the central nervous system, muscles, and the intestinal tract. It also plays a major role in bone metastasis associated with breast, prostate, and lung cancers.^30^ The accumulation of EVs carrying RANKL and the inhibition of EV function using anti-RANKL antibodies may represent important strategies for controlling systemic homeostasis and cancer metastasis.

A recent study using intravital imaging reported that multinucleated osteoclasts on the bone surface fissure into smaller daughter cells, called osteomorphs, are recycled and fused into larger osteoclasts in response to soluble RANKL administration.^31^ In addition, during suppression of RANKL signaling by OPG-Fc administration, these daughter cells are accumulated and re-fused after OPG administration is interrupted.^31^ It is possible that such recycling of osteomorphs, along with the accumulation of osteoclast precursors, may contribute to the overshoot of bone resorption observed in mice after the discontinuation of anti-RANKL antibody treatment in the present study. In addition, in this study, expansion of hematopoietic stem and progenitor cells, including HSCs, CLPs, and CMPs as well as osteoclast progenitors, was observed during anti-RANKL antibody treatment. Thus, inhibition of RANKL signaling not only suppresses the bone-resorbing function of osteoclasts but also has an impact on the wide branches of hematopoiesis in the bone marrow environment.

The present study also demonstrated that inhibition of RANKL signaling by anti-RANKL antibody reduced the number of osteoblast progenitor cells, resulting in decreased bone formation. As shown in Fig. S7e, direct stimulation of RANKL on osteoblast by anti-RANKL antibody did not affect osteoblast differentiation. What is the mechanism underlying the long-term suppression of bone formation? During treatment with anti-RANKL antibody, RANKL-bearing EVs and osteoclast precursors accumulated; after discontinuation of treatment, the accumulated Exploring mechanisms of anti-RANKL treatment discontinuation bearing EVs promoted osteoclastogenesis, resulting in an overshoot phase. During the overshoot period, RANKL-bearing EVs stimulate osteoclastogenesis, while RANK-bearing EVs derived from osteoclasts may increase. It has been reported that RANK-containing EVs promote bone formation via reverse signaling through interaction with RANKL expressed on osteoblasts,^32^ which does not explain the long-term suppression of bone formation after discontinuation. On the other hand, it has also been reported that RANK signaling on BMSCs inhibits osteoblast differentiation from BMSCs, including PαS cells.^33^ However, considering that the overshoot period is a transient phenomenon that recovers to normal levels within 2 weeks of the peak period, it is unlikely that RANKL- and RANK-bearing EVs are involved in the long-term suppression of bone formation thereafter. A recent clinical study found that bone biopsies from patients treated with denosumab showed a higher number of empty osteocyte lacunae, indicating a lack of viable osteocytes during denosumab treatment; this elevated number of empty lacunae remained high after denosumab discontinuation.^14^ In addition, single-cell spatiotemporal analysis and lineage-tracing studies of bone formation under physiological and pathological conditions have revealed the details of the origin, identity, and fate of osteoblasts.^34,35^ Considering the limited analysis of osteogenic progenitor cells in this study, future research using such advanced strategies may reveal the mechanisms by which RANKL antibodies reduce BMSCs, osteoblasts, and even osteocytes. However, the finding that G-CSF levels were elevated in mice treated with anti-RANKL antibodies may explain the disruption of bone metabolism in these mice.

G-CSF is widely used clinically to mobilize HSCs from the bone marrow niche to the peripheral bloodstream before transplantation. G-CSF directly regulates the proliferation and differentiation of HSCs, resulting in the expansion of phenotypic HSCs, including LSK cells. G-CSF also acts on MSCs to suppress the differentiation of pre-osteoblasts, while simultaneously downregulating the expression of stromal cell-derived factor 1, which is essential for maintaining HSCs within their niche in the bone marrow.^36^ Furthermore, G-CSF strongly inhibits osteoblast function and also causes the ablation of mature osteoblasts.^37,38^ Thus, G-CSF is associated with dynamic changes in the bone marrow microenvironment, primarily by decreasing bone volume. Future studies are needed to shed more light on the mechanism underlying bone deterioration that occurs after the discontinuation of anti-RANKL treatment by clarifying the broader impact of increased G-CSF expression on the osteoblast lineage, including MSCs. Taken together, a better understanding of the functions of RANKL in directly regulating the bone marrow environment, such as EV clearance and maintenance of hematopoietic and mesenchymal bone marrow cells, may provide insights into to the specific bone loss that occurs after anti-RANKL antibody discontinuation.

Patients with osteoporosis require lifelong medical care; therefore alternative treatments should be recommended if denosumab therapy is discontinued. However, in studies on switching medication after long-term denosumab use, bisphosphonate treatment did not completely prevent bone loss and fracture.^39^ The primary pathogenesis of bone loss and increased risk of fracture after discontinuation of anti-RANKL antibody therapy would be the long-term suppression of bone formation uncoupled with a rapid increase in bone resorption. Our findings, though limited, provide a molecular basis for advancing long-term therapeutic strategies and highlight the challenges of discontinuing denosumab.

## Methods

### Study design

The main objective of this study was to investigate whether and how an osteoclastogenic burst is induced after discontinuation of denosumab in patients with osteoporosis. To this end, we examined the bone architecture and metabolism in mice after discontinuation of anti-RANKL injection by focusing on both osteoblasts and osteoclasts. Then we analyzed the progenitors of osteoblasts and osteoclasts in mice after discontinuation. We also assessed the effects of serum taken from mice after discontinuation on osteoclastogenesis. To identify what signals osteoclastogenic burst, we determined the effect of EVs isolated from patients and mice after discontinuation of anti-RANKL treatment on osteoclastogenesis. Finally, we found that the anti-RANKL antibody inhibited the clearance of EVs by macrophages. All “replication” referred to in this study means biological replicates. Data were acquired and analyzed in a blinded manner and are from at least three independent experiments. The exact number of experiments and P value are provided in the respective figure legends.

### Ethics

This study was approved by the institutional research committee of Showa University and Yamanashi Red Cross Hospital and conducted in accordance with the Declaration of Helsinki.

### Mice and administration of anti-RANKL antibody

All animals were maintained under specific pathogen-free conditions, and all experiments were performed with approval of the Institutional Animal Care and Use Committee of Showa University (Tokyo, Japan). C57BL/6J and C57BL/6-Tg (CAG-EGFP) mice were purchased from CLEA Japan, Inc. (Shizuoka, Japan) and Japan SLC, Inc. (Shizuoka, Japan), respectively. To generate an experimental model of denosumab discontinuation, a rat anti-mouse-RANKL antibody (clone OYC1; ORIENTAL YEAST Co., Ltd., Tokyo, Japan) was used because denosumab is not cross-reactive with rodent RANKL. Eight-week-old female mice were subcutaneously injected with 5 mg/kg anti-RANKL antibody or saline. The bisphosphonate discontinuation group was subcutaneously injected with risedronate (5 μg/kg) every other day for 4 weeks followed by treatment discontinuation.

### Bone analyses

The BMD of the lumber spine and femoral neck in patients was evaluated using a dual-energy x-ray absorptiometry machine. The changes in bone volume in mice during the experimental periods were analyzed every 2 weeks using an in vivo three-dimensional (3D) micro-CT system (R_mCT2; Rigaku Co., Tokyo, Japan). Changes in bone mass were assessed using images of the coronal section of the proximal femur using ImageJ 1.44p software (National Institutes of Health, Bethesda, MD, USA). For detailed analysis of the bone structure, the femur and L5 vertebra were scanned using the ScanXmate-L090H system (Comscantecno Co., Ltd., Yokohama, Japan), and 3D images were reconstructed with the TRI/3D-Bon-FSC system (RATOC System Engineering, Santa Clara, CA, USA) as previously described.^26^ Bone strength was analyzed with a three-point bending test of the tibia. To assess the chemical composition of the bone, FTIR images of cortical bone of the femur were acquired using the Spectrum Spotight 400 Imaging System (PerkinElmer, Waltham, MA, USA) as previously described.^40,41^ For bone morphometric analysis, tibiae were dehydrated and embedded in glycol methacrylate, sectioned into 3-μm-thick longitudinal sections with a microtome, and stained with toluidine blue or TRAP. For double labeling experiments, mice were injected intraperitoneally with calcein at a dose of 25 mg/kg body weight at 4-day intervals before sacrifice. Tibiae were then obtained, dehydrated, embedded in glycol methacrylate, and sectioned into 3-µm-thick longitudinal sections using a microtome. Static and dynamic parameters of bone resorption and formation were measured using the OsteoMeasure Bone Histomorphometry System (OsteoMetrics, Atlanta, GA, USA) as previously described.^26^

### Measurement of biomarkers and cytokines

Serum levels of TRAP, RANKL, and OPG were determined using a Mouse TRAP™ ELISA Kit (Immunodiagnostic Systems, East Boldon, UK), Mouse TRANCE/RANKL/TNFSF11 Quantikine ELISA Kit (R&D systems, Minneapolis, MN, USA), and Mouse Osteoprotegerin/TNFRSF11B Quantikine ELISA Kit (R&D systems), according to the manufacturer’s instructions. Serum levels of ALP were determined using a LabAssay (Wako Chemicals, Osaka, Japan), which utilizes the substrate p-nitrophenyl phosphatate. The concentrations of mouse chemokines were measured using the Bio-Plex Pro Mouse Cytokine Assay (#M6009RDPD; Bio-Rad Laboratories, Inc., Hercules, CA, USA) to assess the following parameters: eotaxin, G-CSF, IFN-γ, IL-1α, IL-1β, IL-3, IL-4, IL-5, IL-6, IL-9, IL-10, IL-12 (p40), IL-12 (p70), KC, IL-17A, MCP-1, MIP-1α, MIP-1β, RANTES, and TNF-α.

### Quantitative PCR

To measure *Acp5* and *Ctsk* mRNA expression, total mRNA was purified from bone marrow cells collected from the tibia and femur. For *Tnfsf11* mRNA expression, total mRNA was purified from the whole tibia and femur without removing bone marrow cells. Quantitative PCR (qPCR) was performed using the QuantStudio3 Real-Time PCR System with PowerUp SYBR Green Master Mix (Applied Biosystems, Foster City, CA, USA), according to the manufacturer’s protocol. The level of mRNA expression was normalized to that of *Gapdh*. The following primers were used: *Acp5*, 5′-ttgcgaccattgttagccac-3′ (sense) and 5′-cacaccgttctcgtcctgaa-3′ (antisense); *Ctsk*, 5′-tgtgaaccatgcagtgttgg-3′ (sense) and 5′-ggcgttgttcttattccgagc-3′ (antisense); *Tnfsf11*, 5′-gcagaaggaactgcaacaca-3′ (sense) and 5′-gatggtgaggtgtgcaaatg-3′ (antisense); and *Gapdh*, 5′-aactttggcattgtggaagg-3′ (sense) and 5′-ggatgcagggatgatgttct-3′ (antisense).

### Osteoblast differentiation and CFU assays

In vitro osteoblast differentiation was performed as previously described.^42^ Briefly, osteoblast progenitor cells derived from the mouse calvarial bone of newborn mice were cultured in osteogenic medium (50 μmol/L ascorbic acid, 10 nmol/L dexamethasone, 10 mmol/L β-glycerophosphate), after which ALP activity (after 7 days) and bone nodule formation (after 21 days) were analyzed. ALP activity was evaluated by ALP staining and bone nodule formation by Alizarin Red S staining. For the CFU assay, bone marrow cells were cultured in Alpha-Minimum Essential Medium (α-MEM) containing 10% FBS for 3 days, followed by culture in osteogenic medium with medium changes every 3 days. Anti-RANKL antibody was added 2 days after changing to osteogenic medium. CFU-ALP and CFU-Ob were detected as ALP-positive colonies and Alizarin Red S-positive colonies, respectively.

### Osteoclast differentiation

In vitro osteoclast differentiation was performed as previously described.^43^ Briefly, nonadherent bone marrow cells from the mouse femur were cultured in α-MEM with 10% FBS containing 10 ng/mL macrophage CSF (M-CSF) (R&D Systems) for 2 days to obtain BMMs. These cells were further cultured for 3 days with 50 ng/mL RANKL (R&D Systems) in the presence of 10 ng/mL M-CSF with 10% FBS. Human PBMCs were isolated using a Ficoll gradient and cultured in the same manner as osteoclastogenesis from the mouse BMMs. Osteoclastogenesis was evaluated by counting TRAP-positive multinucleated (more than 3 nuclei) cells.

### Flow cytometry analysis

For analysis of bone marrow-derived osteoclast precursor cells, a single-cell suspension of mouse bone marrow cells was stained with the following antibodies; PerCP-Cy5.5 anti-CD3 (17A2; BioLegend, San Diego, CA, USA), PerCP-Cyanine5.5 anti-CD45R (B220) (RA3-6B2), Super Bright 436 c-Kit (2B8), APC CD115 (c-fms) (AFS98), PE-Cyanine7 anti-CD11b (M1/70), and Alexa Fluor 488 anti-Ly6C (HK1.4; eBioscience, Inc., San Diego, CA, USA). Dead cells were excluded as Zombie Aqua (BioLegend)-stained cells. For analysis of osteoblast progenitor cells, a single-cell suspension of mouse bone marrow cells and bone cells collected by collagenase and DNase treatment was stained with following antibodies: Zombie Aqua, FITC anti-TER-119/Erythroid Cells (TER-119, BioLegend), FITC anti-CD45 (30-F11), APC anti-Sca-1 (D7), Biotin anti-CD140a (PDGFRA) (APA5, eBioscience), Brilliant Violet 421 Streptavidin (BioLegend), goat anti-mouse ALPL polyclonal antibody, and PE anti*-*goat IgG (Invitrogen). Flow cytometry analysis was performed using the LSRFortessa flow cytometer (BD Biosciences, Franklin Lakes, NJ, USA) and analyzed with FlowJo software (BD Biosciences).

### Isolation of EVs and phagocytosis assay

EVs were isolated from the pooled serum of mice or untreated patients by differential ultracentrifugation as previously described.^44^ In brief, serum was centrifuged at 2 000 × *g* for 10 min at 4 °C followed by ultracentrifugation at 10 000 × *g* for 30 min to separate the pellet containing apoptotic bodies from the supernatant. Then the supernatant was ultracentrifuged at 30 000 × *g* for 70 min at 4 °C and the pellet containing the microvesicles and supernatant was separated. The supernatant was further ultracentrifuged at 100 000 × *g* for 70 min at 4 °C to collect the final supernatant lacking EVs, and the exosome-containing pellet was collected after washing with further ultracentrifugation at the same speed. To examine the effects on osteoclastogenesis, the EVs isolated from 1 mL mouse or human serum were added to mouse BMM or human PBMC cultures in the presence of M-CSF and RANKL, respectively. The effect of mouse or human EV-depleted serum on osteoclastogenesis was tested by dilution with medium to 5% or 10%, respectively. For the phagocytosis assay, fluorescent EVs were isolated from the serum of C57BL/6-Tg (CAG-EGFP) mice after incubation with anti-RANKL antibody (OYC1, 100 μg/mL) or isotype control IgG for 2 h and then added to BMMs.

### Immunoblot analysis and immunogold labeling of EVs

Total protein (10 μg) extracted from the pellet containing ABs, MVs, or exosomes was subjected to immunoblot analysis using rabbit anti-CD63 (System Biosciences, Palo Alto, CA, USA) and mouse anti-RANKL (G-1; Santa Cruz, Dallas, TX, USA) as the primary antibodies; horseradish peroxidase (HRP)-conjugated anti-rabbit IgG and HRP-conjugated anti-mouse IgG as the secondary antibodies; and the ECL Plus Western Blotting Detection system (GE Healthcare, Chicago, IL, USA). For scanning electron microscopy (S-4700 electron microscope; Hitachi, Tokyo, Japan), EVs were immunolabeled with an anti-RANKL antibody (C30535; Assay Biotechnology Co., Inc., San Jose, CA, USA) or isotype control IgG (2729; Cell Signaling Technology, Danvers, MA, USA) followed by staining with 6 nm colloidal gold-conjugated anti-rabbit secondary antibody (Jackson ImmunoResearch Labs, West Grove, PA, USA).

### Statistical analyses

Statistical analyses were performed using the unpaired two-tailed Student’s *t* test or one-way analysis of variance (ANOVA) (*P < 0.05; **P < 0.01; ***P < 0.001). When ANOVA indicated a significant difference, the specific difference was calculated with the Holm-Sidak post-hoc test. Data are expressed as the mean ± standard error of the mean (SEM). Results are presented as representative examples of more than three independent experiments.

## Supplementary information

Supplemental material
