# Supplementary material for: Mouse model of anti-RANKL discontinuation reveals reduced bone mass and quality through disruption of bone remodeling
Source: Bone Res. 2025 May 28;13:56. doi: 10.1038/s41413-025-00433-0 (PMC12116787; doi:10.1038/s41413-025-00433-0)
Supplement: Supplementary file 1 — Supplemental material [file 41413_2025_433_MOESM1_ESM.docx]

**Supporting Information**

**Mouse model of anti-RANKL discontinuation reveals reduced bone mass and quality through disruption of bone remodeling**

Koji Ishikawa^1,2^, Soji Tani^1^, Nobuhiro Sakai^3^, Yoshifumi Kudo^1^, Hideyo Horiuchi^4^, Hiromi Kimura-Suda^5^, Masamichi Takami^6^, Mayumi Tsuji^2^, Katsunori Inagaki^1^, Yuji Kiuchi^2^, Takako Negishi-Koga^1,7,8,9^*

*Corresponding author: Takako Negishi-Koga (T. N-K.)

Department of Pathophysiology for Locomotive Diseases, Juntendo University Graduate School of Medicine, Tokyo, Japan

Address: 2-1-1, Hongo, Bunkyo-ku, 113-8421, Tokyo, Japan

Email: [t.negishi.ob@juntendo.ac.jp](mailto:t.negishi.ob@juntendo.ac.jp)

**Supporting Information includes:**

Figure S1 to S8

Table S1 and S2


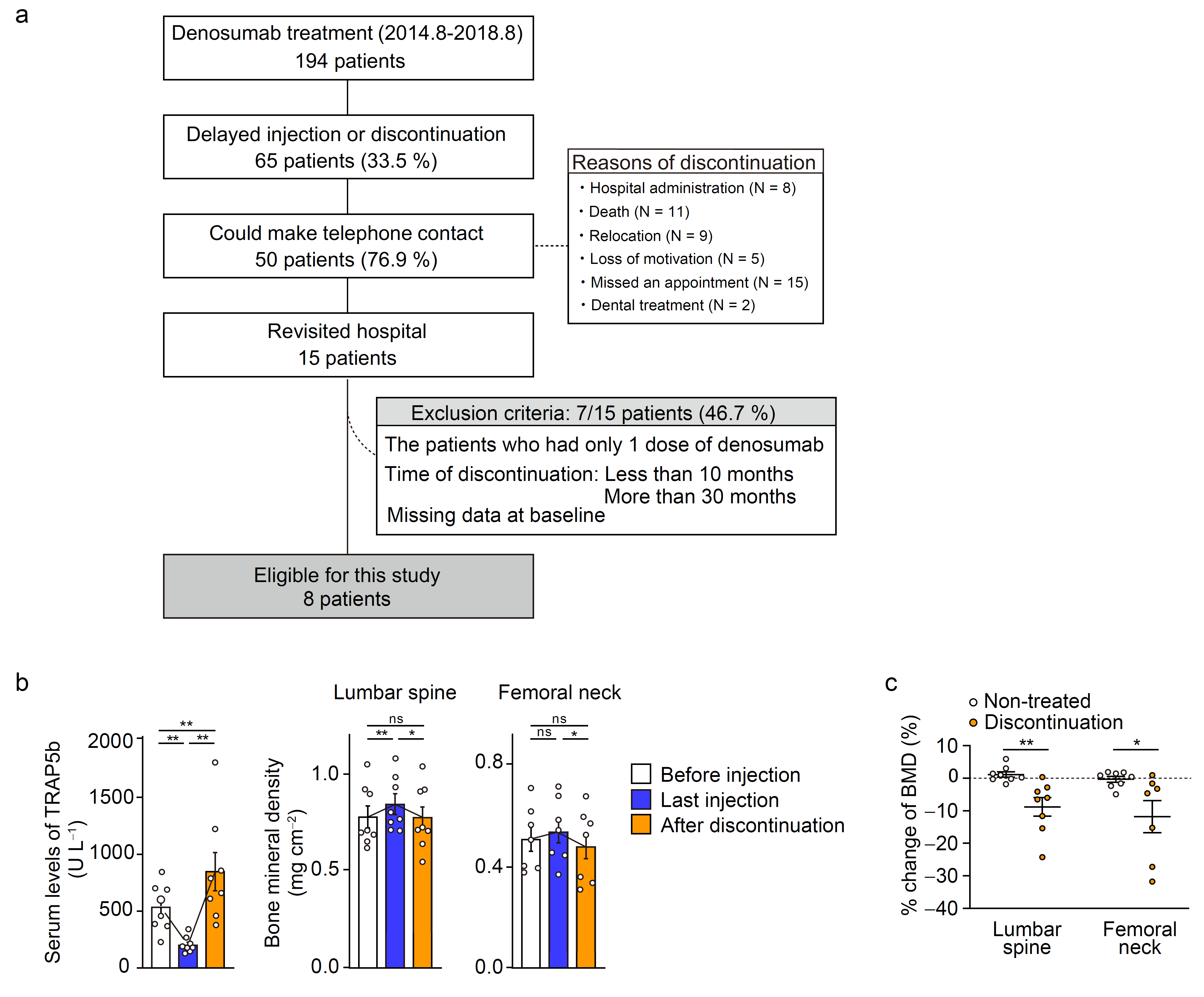


**Fig. S1. TRAP overshoot in patients after discontinuation of denosumab treatment**

**a** Flow diagram of eligible patients. A total of 194 patients with postmenopausal osteoporosis who received multiple anti-RANKL antibody injections between August 2015 and August 2018 were screened. After meeting the inclusion criteria, eight patients were enrolled in this study. Age- and sex-matched untreated patients who had undergone at least two DXA scans were included as the control group. **b** Serum levels of TRAP5b and the BMD of the lumber spine and femoral neck in patients before anti-RANKL treatment, after the last dose, and after discontinuation (n = 8).  The mean number of injections is 4.4 times. The mean interval from 'Before Injection' to 'Last Injection' and from 'Last Injection' to 'After Discontinuation' is 26.3 months and 17.1 months, respectively. Data are presented as the mean ± SEM. *: *p* < 0.05, **: *p* < 0.01, ns: not significant. Wilcoxon test was performed. **c** Percent change in BMD after discontinuation of denosumab treatment (n = 8) compared to the non-treated patients (n = 8) (The femoral neck BMD analysis of the discontinuation group included seven patients due to one patient's bilateral hip fracture). The mean interval of DXA assessments for the Non-treated and Discontinuation groups is 19.9 months and 17.1 months, respectively. Data are presented as the mean ± SEM. *: *p* < 0.05, **: *p* < 0.01. Mann-Whitney U test was performed.


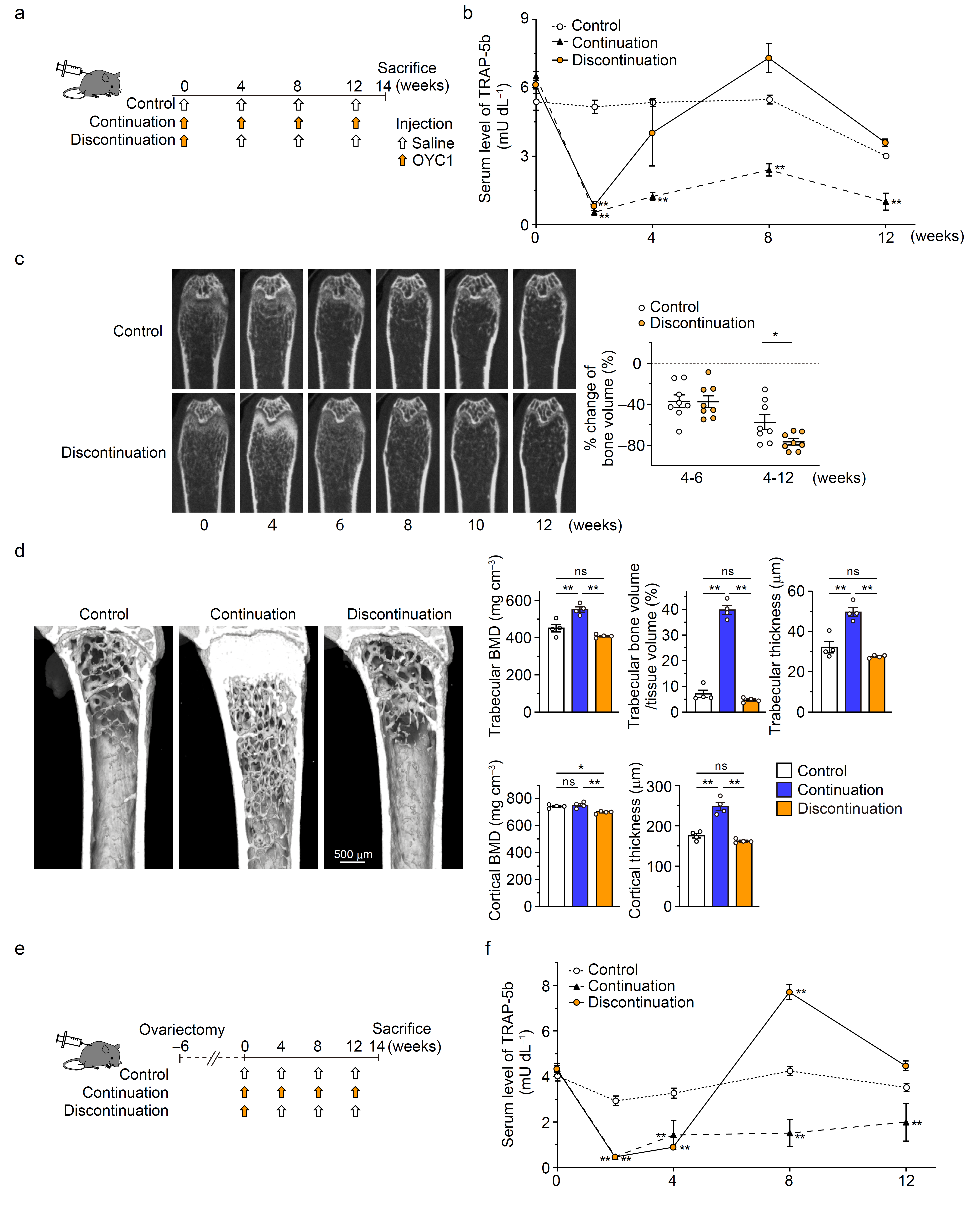
 **Fig. S2. Bone metabolism in mice after discontinuation of single-doze of anti-RANKL antibody (Single-injection model) and ovariectomized mice after discontinuation of single-doze of anti-RANKL antibody (OVX plus single-injection model)**

**a** Schematic of the experimental setting for the mouse model of anti-RANKL discontinuation (Single-injection model). **b** Serum TRAP levels over time in mice that continued to receive anti-RANKL antibody (continuation), mice that stopped receiving it (discontinuation), and control mice (n = 5). **c** Representative *in vivo* CT images of the femur in discontinuation and control mice (left), and the percent change in bone volume (right) (n = 8). **d** Representative μCT images of the femur metaphysis (left) and parameters of the distal femur at 14 weeks (n = 4). **e** Schematic of the experimental setting for the single-injection model of anti-RANKL discontinuation in ovariectomized mice. **f** Serum TRAP levels over time in ovariectomized mice that continued to receive anti-RANKL antibody (continuation), mice that stopped receiving it (discontinuation), and control mice (n = 5). All values are representative of at least three independent experiments and are displayed as the mean ± SEM. *: *p* < 0.05, **: *p* < 0.01, ns: not significant. Holm-Sidak post hoc test (**b, d,** **f**) and Student’s *t*-test (**c**) were performed.

**
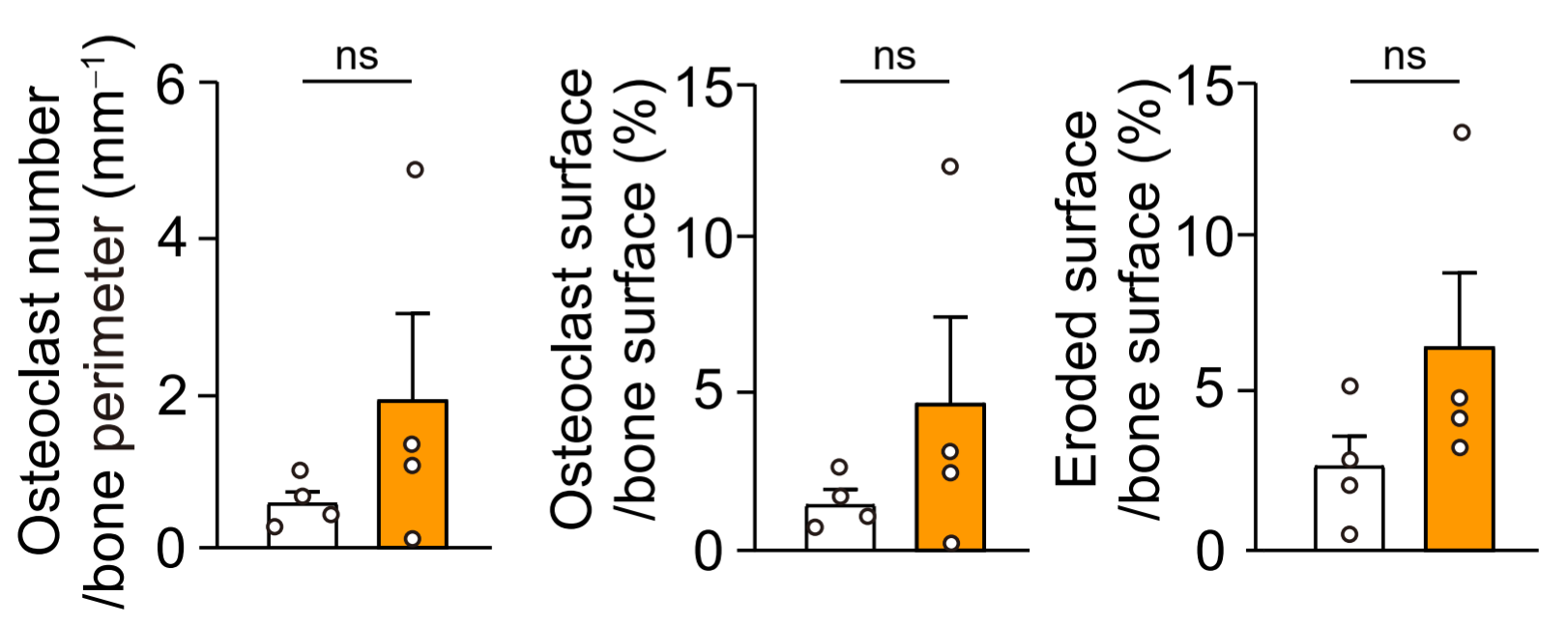
**

**Fig. S3. Bone morphometric parameters of bone resorption in mice long after discontinuation of anti-RANKL antibody (Three-injection model)**

Parameters for osteoclastic bone resorption determined by bone histomorphometric analysis. All values are representative of at least three independent experiments and are displayed as the mean ± SEM. ns: not significant. Student’s *t*-test was performed.

**
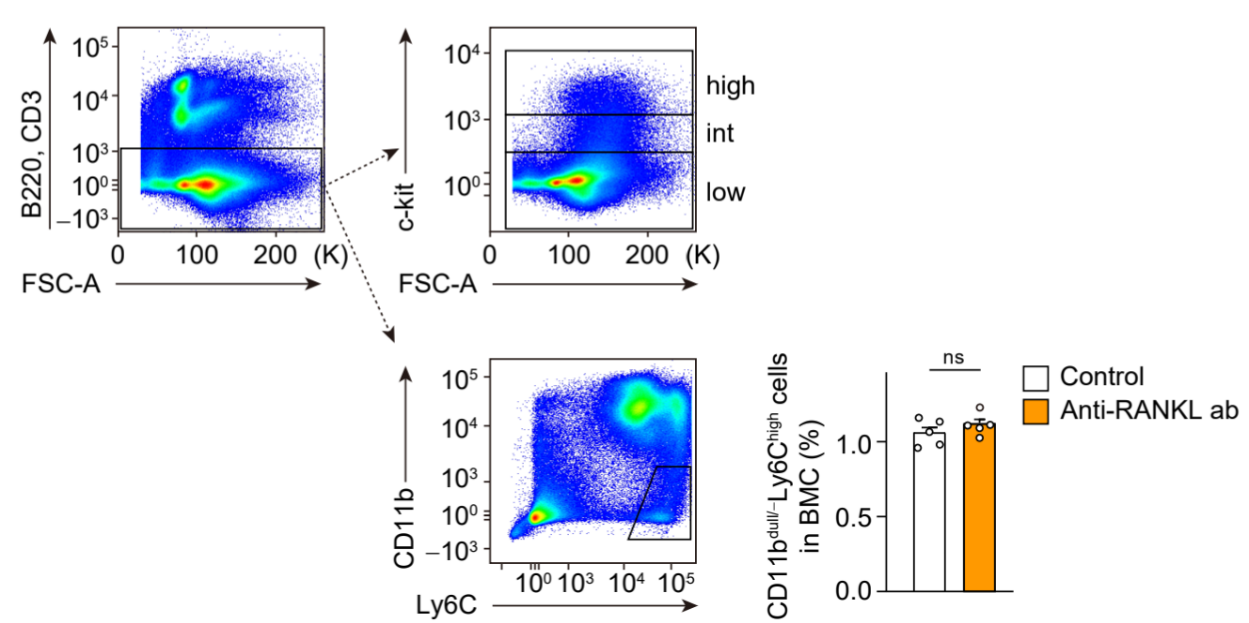
**

**Fig. S4. Gating strategy to distinguish osteoclast precursor cells and comparison of osteoclast precursors between anti-RANKL discontinuation and bisphosphonate discontinuation**

**a** Gating strategy to distinguish subpopulation characterized with c-kit expression. The osteoclast precursor cells were examined in the subpopulation gated by c-kit (high, int and low) or the population gated by CD11b^dull/−^ and Ly6C^high^. The percentage of B220^−^CD3^−^CD11b ^dull/−^Ly6C^high^ cells in BMCs of anti-RANKL antibody-treated mice 2 weeks after the last injection (three-injection model) (right, n = 5). **b** The percentage of osteoclast precursor cells in anti-RANKL antibody discontinuation mice, bisphosphonate discontinuation mice and control mice 2 weeks after the last injection (n = 5). All data are representative of at least three independent experiments and are displayed as the mean ± SEM. ***: *p* < 0.001, ns: not significant. Student *t*-test (**a**) and one-way ANOVA (**b**) were performed.


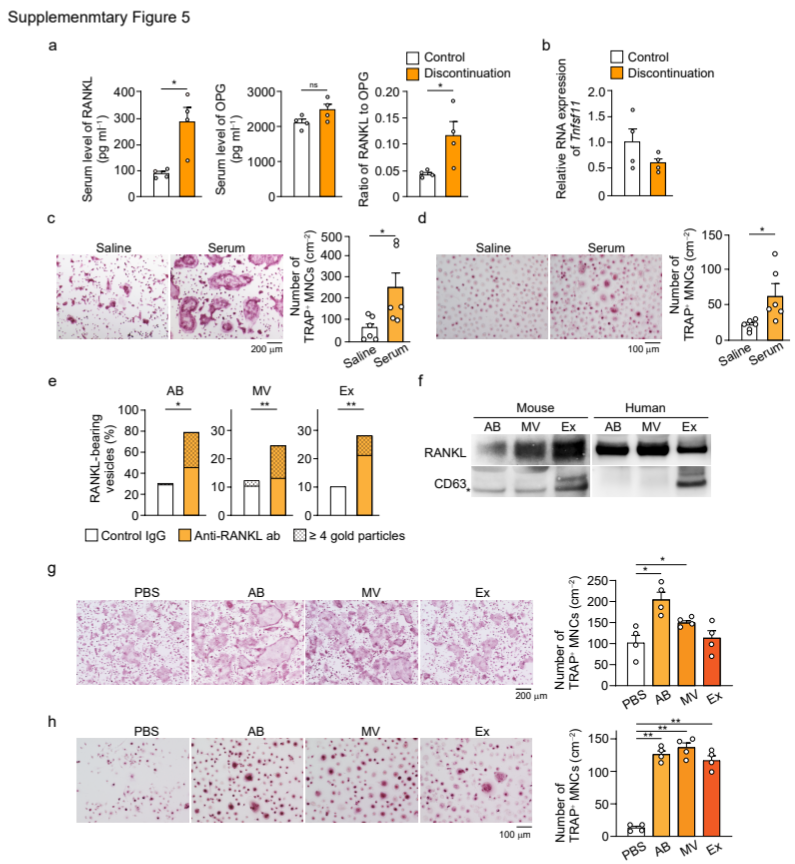


**Fig. S5. Effect of RANKL-bearing extracellular vesicles on osteoclastogenesis**

**a** Serum levels of RANKL and OPG levels in control mice and mice with treatment discontinuation during the overshoot period at 8 weeks after the last injection (three-injection mouse model) (n = 4). **b** Relative mRNA expression of Rankl (encoded by Tnfsf11) in the femur of control mice and mice with treatment discontinuation at 8 weeks after the last injection (n = 4). **c** Representative images of osteoclast differentiation of BMMs cultured with serum (left). Number of TRAP-positive multinucleated cells (TRAP^+^ MNCs) (right) (n = 6). **d** Representative images of osteoclast differentiation of PBMCs cultured with serum (left). Number of TRAP^+^ MNCs (right) (n = 6). **e** Ratio of RANKL-bearing vesicles in the apoptotic bodies (AB), microvesicles (MV), and exosomes (Ex) isolated from normal mouse serum. **f** RANKL expression in EVs isolated from mice and human serum. *: non-specific. **g** Representative images of osteoclast differentiation of BMMs cultured with the EVs isolated from non-treated patients (left). Number of TRAP^+^ MNCs (right) (n = 4). **h** Representative images of osteoclast differentiation of PBMCs cultured with the EVs isolated from non-treated patients (left). Number of TRAP^+^ MNCs (right) (n = 4). All values are representative of at least three independent experiments and are displayed as the mean ± SEM. *: *p* < 0.05, **: *p* < 0.01. Student’s *t*-test (a, b, **c** and **d**), χ^2^ test (**e**) and Dunnett’s test (**g** and **h**) were performed.


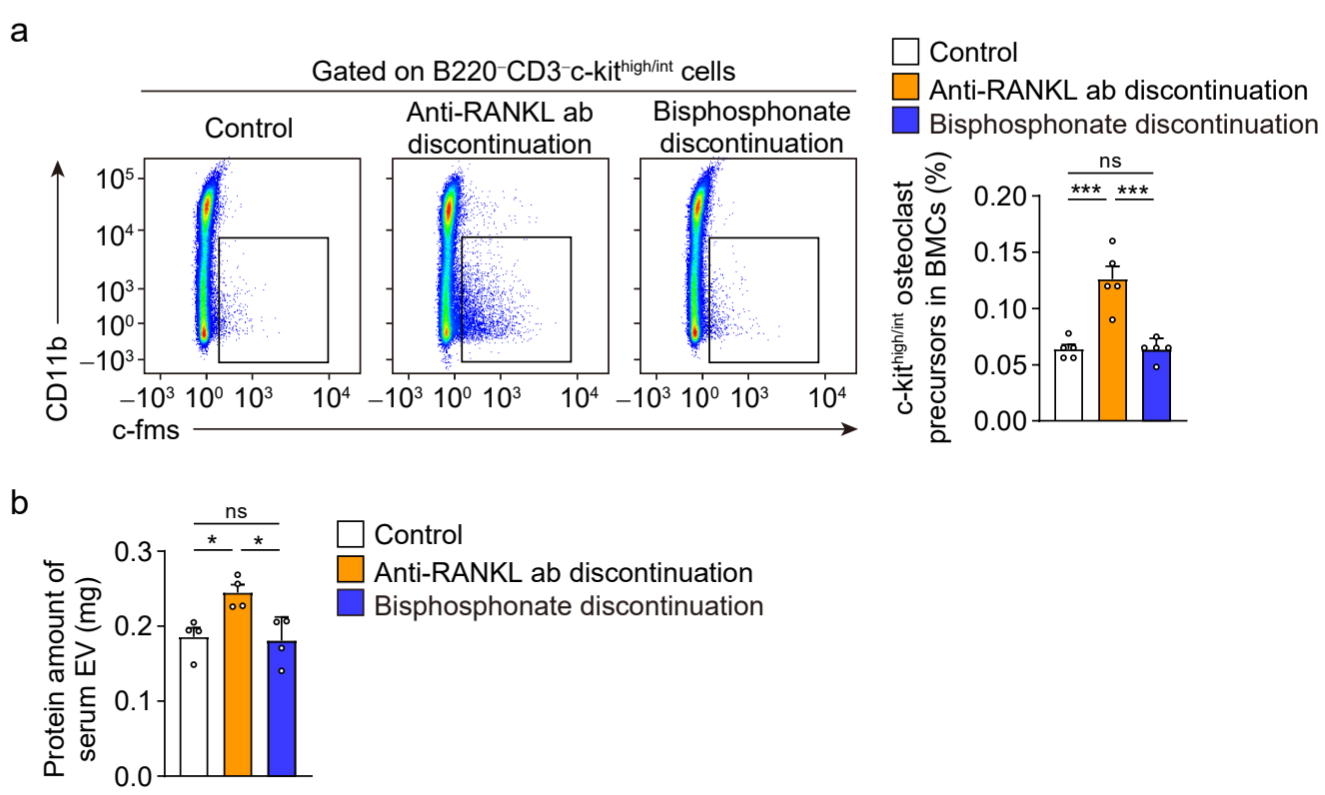


**Fig. S6. Comparison of the effects of discontinuing anti-RANKL antibody treatment and discontinuing bisphosphonate treatment on osteoclast precursors and EV amount**

**a** The percentage of osteoclast precursor cells in anti-RANKL antibody discontinuation mice, bisphosphonate discontinuation mice and control mice 2 weeks after the last injection (n = 5). **b** Protein amount of serum EVs in the control, anti-RANKL antibody-treated, and risedronate (bisphosphonate)-treated mice 10 weeks after the last injection (n = 5). All data are representative of at least three independent experiments and are displayed as the mean ± SEM. *: *p* < 0.05, ***: *p* < 0.001, ns: not significant. One-way ANOVA were performed.


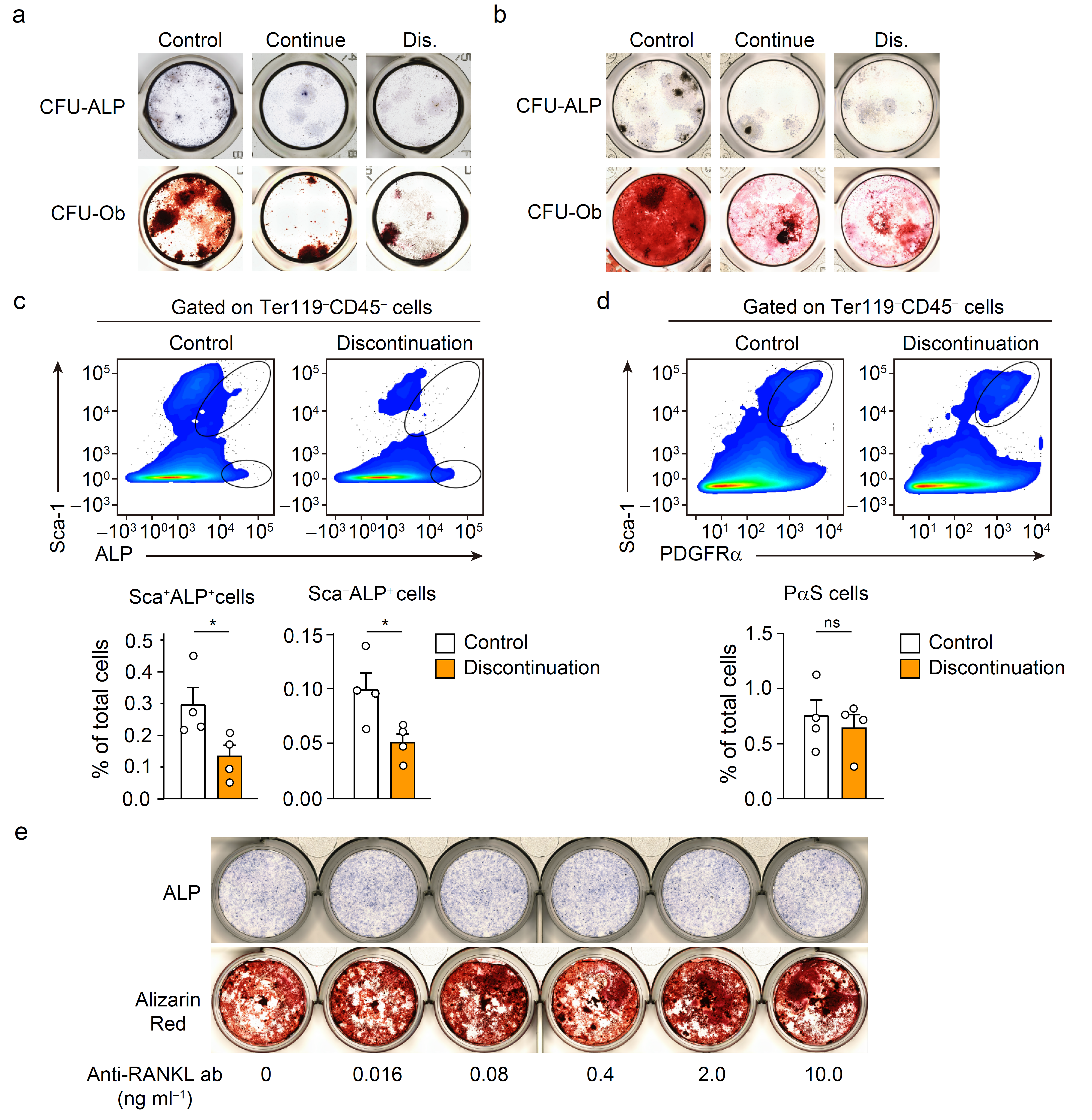


**Fig. S7. Suppression of osteoblast lineage cells after discontinuation**

**a,** Representative images of the generation of alkaline phosphate (ALP) positive colony forming units (CFU-ALP) and alizarin-red positive CFUs (CFU-Ob) in bone marrow cells (BMCs) derived from control mice and mice injected with single-dose of anti-RANKL antibody 14 weeks after injection (n = 6). **b** Representative images of the generation of CFU-ALP and CFU-Ob in BMCs derived from ovariectomized mice injected with single-dose of anti-RANKL antibody 16 weeks after injection (n = 6). **c, d** Flow cytometric analysis of BMCs derived from the OVX plus three-injection model mice at 16 weeks after discontinuation (n = 4). The percentage of osteoblastic precursors characterized by the cell surface markers Ter119^−^CD45^−^ALP^+^Sca-1^+^ cells (**c**), Ter119^−^CD45^−^ALP^+^Sca1^−^ cells (**c**,) and Ter119^−^CD45^−^Sca-1^+^PDGFRα^+^ (**d**). **e** Osteoblast differentiation and bone nodule formation in calvarial cell-derived osteoblasts. All values are representative of at least three independent experiments and are displayed as the mean ± SEM. *: *p* < 0.05, **: *p* < 0.01. Student’s *t*-test was performed.

**Table. S1. Comparison of baseline patient characteristics with anti-RANKL treatment discontinuation and age- matched control.**

| **Parameters** | **Control** | **Discontinuation** | ***p*** |
| --- | --- | --- | --- |
| Age (years) | 74.1±6.2 | 79.9±5.4 | n.s. |
| Serum Ca (mg/dL) | 9.3±0.3 | 9.3±0.3 | n.s. |
| Serum P (mg/dL) | 3.5±0.2 | 3.7±0.2 | n.s. |
| Serum Alb (g/dL) | 4.4±0.5 | 4.1±0.3 | n.s. |
| eGFR (mL/min/1.73 m²) | 60.5±12.2 | 55.3±25.2 | n.s. |
| Serum intact-PTH (pg/mL) | 34.9±10.0 | 42.0±17.5 | n.s. |
| Lumber spine-BMD (g/cm²) | 0.76±0.04 | 0.85±0.14 | n.s. |
| Femoral neck-BMD (g/cm²) | 0.60±0.05 | 0.54±0.13 | n.s. |

Comparison of the clinical parameters (n = 8). The data expressed the mean ± SD were analyzed with Student’s t-test. N.s.: not significant, *: p < 0.05, **: p < 0.01. Ca: calcium, p: phosphorus, Alb: albumin, eGFR: estimated glomerular filtration rate, intact-PTH: intact-parathyroid hormone, BMD: bone mineral density.

|  | **Control** | **Discontinuation** | ***P*** |
| --- | --- | --- | --- |
| Eotaxin | 1912.1 ± 243.0 | 2308.2 ± 516.2 | n.s. |
| G-CSF | 73.2 ± 16.8 | 98.2 ± 18.3 | < 0.01 |
| INF-γ | 31.5 ± 9.9 | 37.3 ± 11.9 | n.s. |
| IL-1α | 15.5 ± 2.9 | 15.9 ± 1.7 | n.s. |
| IL-1β | 11.6 ± 7.4 | 17.1 ± 6.9 | n.s. |
| IL-3 | 4.2 ± 2.0 | 6.6 ± 4.6 | n.s. |
| IL-4 | 2.0 ± 1.3 | 2.3 ± 1.2 | n.s. |
| IL-5 | 10.3 ± 4.6 | 13.5 ± 5.7 | n.s. |
| IL-6 | 2.2 ± 2.2 | 6.2 ± 3.9 | < 0.05 |
| IL-9 | 15.5 ± 2.7 | 19.1 ± 9.8 | n.s. |
| IL-10 | 37.1 ± 14.5 | 50.2 ± 16.2 | n.s. |
| IL-12(p40) | 360.0 ± 58.9 | 305.0 ± 107.2 | n.s. |
| IL-12(p70) | 233.4 ± 68.0 | 229.7 ± 35.5 | n.s. |
| IL-17A | 92.7 ± 62.9 | 103.8 ± 41.1 | n.s. |
| KC | 52.8 ± 10.5 | 78.4 ± 35.6 | n.s. |
| MCP-1 | 147.0 ± 35.9 | 182.3 ± 36.5 | n.s. |
| MIP-1α | 4.2 ± 1.4 | 3.6 ± 0.6 | n.s. |
| MIP-1β | 50.9 ± 14.5 | 57.5 ± 13.7 | n.s. |
| RANTES | 65.4 ± 15.5 | 55.2 ± 16.4 | n.s. |
| TNF-α | 106.0 ± 40.7 | 145.9 ± 66.6 | n.s. |

**Table. S2. Production of inflammatory cytokines in mouse serum during the overshoot period (n = 9).**

The data expressed as the mean ± SD (pg ml^−1^) were analyzed with the Student *t*-test. n.s.: not significant
